# Heart Rate Changes Before, During, and After Treadmill Walking Exercise in Normal Dogs

**DOI:** 10.3389/fvets.2021.641871

**Published:** 2021-04-12

**Authors:** Sarah A. Shull, Sarah K. Rich, Robert L. Gillette, Jane M. Manfredi

**Affiliations:** ^1^College of Veterinary Medicine, Michigan State University, East Lansing, MI, United States; ^2^Sportsvet Veterinary Consulting Services, Lancaster, SC, United States

**Keywords:** cardiovascular, monitoring, holter, dog, fitness, auscultation, rehabilitation

## Abstract

In dogs, changes in heart rate (HR) can reflect conditioning, fear, anticipation, and pain; however, these are not routinely assessed in veterinary rehabilitation patients. Knowing the expected HR changes during rehabilitation exercises can guide protocols and can optimize post-operative therapy. The primary objectives of the study were to assess HR in dogs undergoing treadmill exercise (TE) during the walk and to compare the three collection techniques of HR, namely, auscultation, a HR monitor (HR MONITOR), and a Holter monitor (HOLTER). We hypothesized that the HR would increase by 20% during TE, that HR taken after TE would not be the same as the HR during TE, and that all methods of measurement would have good agreement. HR was recorded in all methods simultaneously, in eight adult healthy large breed dogs during rest (REST), immediately before TE (PRE), during TE (WALK), and 15 and 60 s after TE (POST-15, POST-60). Statistical analyses included Spearman and Pearson correlations, Bland-Altman analyses, and a repeated measures ANOVA with Sidak's *post-hoc* test (significant at value of *p* < 0.05). Increased HR was reflected in TE during WALK, and elevations in HR during WALK were not reflected in POST timepoints. Auscultation was also not possible during WALK. Significant moderate-to-strong correlations existed among all monitoring options at each of the timepoints (*rho* range = 0.5–0.9, *p* < 0.05). There were no correlations between peak HR and age or weight. The main limitation of this study is that only healthy and large breed dogs were used. Both monitors captured the increase in HR during exercise and could guide TE regimens to minimize patient risk of injury and to maximize training effectiveness.

## Introduction

Heart rate (HR) has historically been used to assess physical state and the effects of the type and intensity of exercise in the fields of human sports medicine and exercise science (1–3). Auscultation and electrocardiogram (ECG) readings are two traditionally used methods to compile HR data. More recently, HR monitors have become a standard method to acquire HR data, which is used as a training aid for a variety of sports (4–9). In the rehabilitation field, measuring the patient's HR is recommended by the American Physical Therapy Association (APTA) Guide to Physical Therapist Practice (10), although gathering this information may be infrequent and underused (11, 12). HR data can be used during exercise sessions to assess the efforts, cardiovascular status, pain, and recovery speed of the patient.

Therapeutic exercise is commonly used in veterinary rehabilitation (13, 14). While HR during exercise has been examined in athletic dogs, there are gaps in understanding what to expect, and data are lacking for rehabilitation patients (15–23). Swanson et al. (19) developed a visual exertion scale for healthy dogs walking and trotting on a treadmill, finding that an increase in exertion correlated to an increase in HR. The authors recognized that larger studies were needed to validate their subjective measures and that the Holter monitor used in that study may be prohibitively expensive to those in private practice (19). Boddy et al. (20) described collecting HR by auscultation in a 6-min walk test in dogs with congestive heart failure as compared to normal healthy dogs: though post-walk HR did not significantly increase from baseline, distance traveled in the heart failure group was less. Swimmer and Rozanski (21) described using a 6-min walk test, including HR measurement performed in healthy dogs and dogs with pulmonary disease. The HR was measured before the walk but not during the exercise. The dogs were walked up and down a hallway, and not on a treadmill. The technique used to acquire the HR was not described, but it is assumed to be by auscultation as they referred to the study of Boddy et al. (20), and an increase in HR from baseline was seen post-walk in the pulmonary disease group, along with a shorter walk distance.

Although HR has shown to be a sound measurement of physiological status, the information on how the veterinary rehabilitation professional can best obtain and use it is lacking. For clinically useful HR monitoring in a practice setting, the approach must produce valid information, be economical, and be easy to apply and interpret in real time. An additional requirement for rehabilitation practices is the ability of monitoring technique to work while on an underwater treadmill and during exercise. Auscultation using a stethoscope is economical and easy to apply and interpret while the patient is stationary but has not been examined during rehabilitative work. Various Holter monitors have been used in clinical settings on cardiac patients, but their economical use, ease of setup, and ability to assess data in real time during exercise may limit their feasibility in practice (22–25). A HR monitor has been examined previously in dogs under stationary and trot conditions (26, 27); however, the validity and HR range in normal dogs during TE (WALK) using a different waterproof HR monitor model have not been assessed (26, 27). The walk is commonly used in rehabilitation settings, as patients are most often recovering from musculoskeletal or neurologic diseases making a faster pace unsafe. Establishing a normal HR variation and recovery time at the walk is key to tailoring rehabilitation regimens and assessing the progress of the patient. An ideal monitoring system for HR in dogs to be used in the veterinary rehabilitation setting has not been established.

It is also essential to recognize all the relative influences on HR. In dogs, HR has been examined as a physiologic parameter of conditioning, anticipation, fear, and pain, yet it is not routinely assessed in veterinary rehabilitation patients (28–32). Rehabilitative therapies have the potential to elicit stressful responses associated with the anticipation of an exercise or loud noise (treadmill turning on and water rushing in). In the previous studies, stress in the form of loud noises has shown an increased HR in dogs of 15–50 beats per minute (bpm), and stress in the form of anticipation has also elevated HR a mean of 43 bpm (28, 29). HR elevations in these instances suggest a level of stress that may not benefit healing (33–38). In humans, elevated HR has been demonstrated in chronic pain cases, where many of our veterinary rehabilitation patients also experience chronic pain (36, 37). Given that rehabilitation exercises are performed in dogs often with chronic pain or recovering from surgery, distinguishing normal HR elevations and recovery time due to TE from other causes is essential for guiding clinical care to minimize pain and maximize conditioning opportunities.

Knowing the expected normal changes in HR and HR recovery during rehabilitation exercises can guide protocols to optimize therapy and conditioning while monitoring the progress of the patient. Changes in patient pain and stress levels reflected by HR changes can dictate post-operative patient care orders by setting parameters that would indicate the need to decrease the intensity of exercise (by slowing the speed) or its duration. A faster HR recovery is a positive prognostic indicator of recovery in dogs and humans with cardiovascular disease (39–45). Therefore, the primary objectives of the study were to assess the HR in healthy dogs undergoing treadmill exercise (TE) and to compare the feasibility of using each of the three HR data collection techniques, namely, auscultation, a HR monitor, and a Holter monitor, in a practice setting. We hypothesized that HR would increase by 20% from baseline during TE, that HR taken 15 and 60 s after TE would not reflect HR during TE, and that all measurement methods would have good agreement and prove feasible.

## Materials and Methods

### Dogs

This study was compliant with the National Institutes of Health (NIH) guidelines for Humane Care and Use of Animals and was approved by our institutional animal care and use committee with proper consent forms filled out. Eight client-owned dogs were used, with three intact males (one German Wirehaired Pointer, one Curly-Coated Retriever, and one Labrador Retriever), one neutered male (mixed breed), one intact female (Labrador Retriever), and three spayed females (Curly-Coated Retrievers). The average age of the dogs was 52 (SD ± 46) months (with all dogs being between 7 months and 10 years of age), weight was 29.1 (SD ± 3.7) kg, and body condition score was 4–5/9. All dogs were previously acclimated to the treadmill and treadmill room and had completed three 6-min walks on the treadmill within 3 months prior to the start of this study. Dogs were examined and found to be generally healthy, based on a general, orthopedic, and neurologic examination performed by the veterinarian who is in charge of the rehabilitation unit of the hospital. None of the dogs had a history or current evidence of cardiovascular, neurologic, or musculoskeletal disease. The medical records for the dogs were available, and none of the dogs had a previous diagnosis of osteoarthritis or other musculoskeletal diseases which would interfere with this study. The regular exercise routine of the dog included walking for 1.6 km for ~15 min, four times a week, for 3 months before the start of the study. No specific conditioning training was performed for this study.

### Heart Rate Monitors and Placement

**Figure 1:**
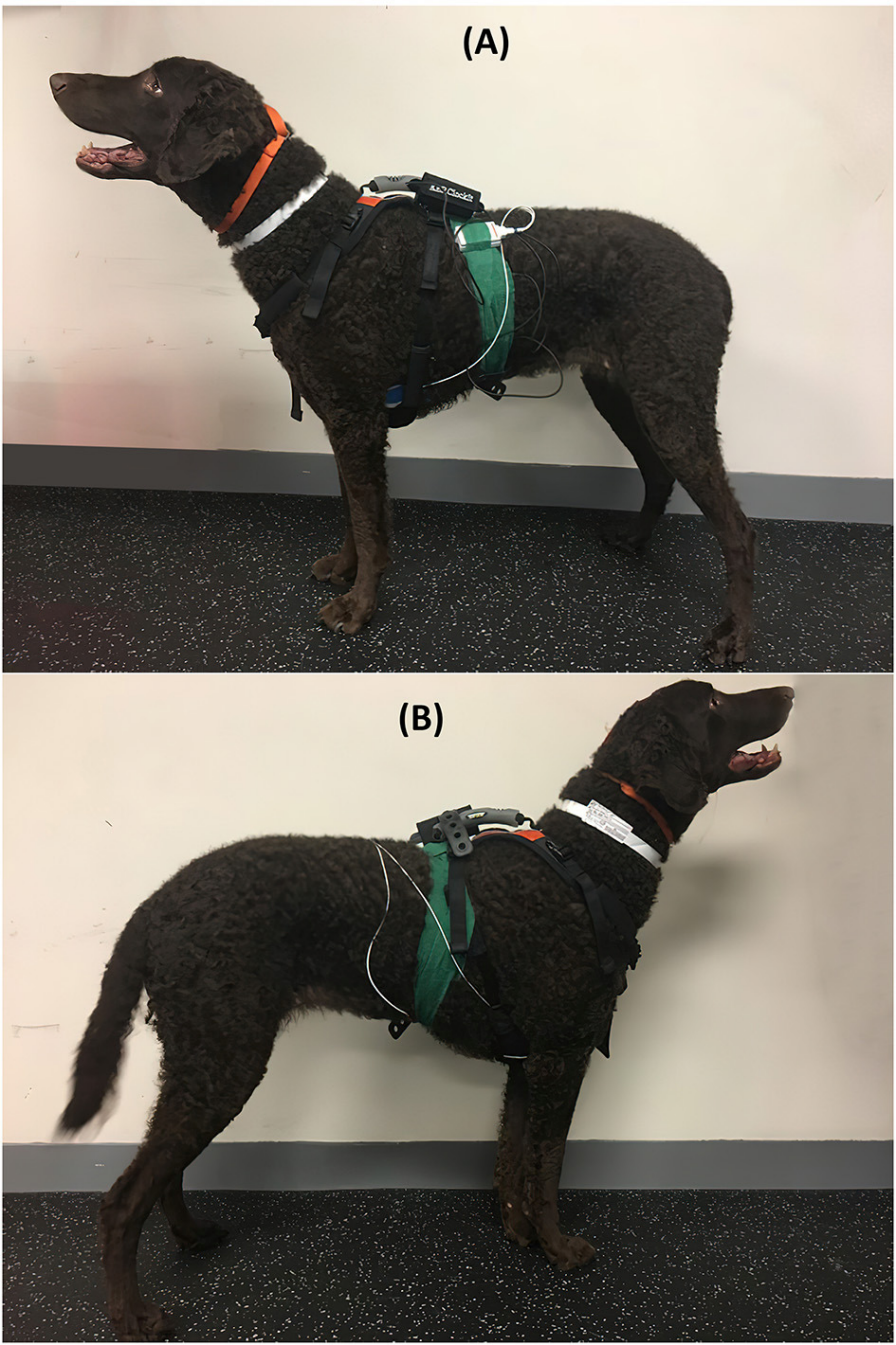
Dog equipped with both the HOLTER and HR MONITOR monitors from the left side **(A)**, where both the HOLTER and HR MONITOR monitors have pads, and from the right side **(B)**, where only HOLTER has pads.

A Holter monitor (HOLTER; Dextronix, Sacramento, CA, USA) and a HR monitor (HR MONITOR; Polar USA, Bethpage, NY, USA) were used to assess HR in addition to auscultation with a stethoscope. Both leads and pads of the HOLTER and the HR MONITOR were waterproof, with their electronics completely encased in plastic so water would not interfere with readings (46, 47). For HOLTER placement, a 3 × 3 cm area of hair was clipped, and the skin was cleaned with alcohol in three areas, one over the sternum and one each over the ventral left and right thoraces, before each of the three HOLTER ECG pads was applied. The hair caudal to the HOLTER on the ventrolateral aspect on the left chest of the dog was wet with alcohol before the placement of the single HR MONITOR pad. Both took 5 min to place in total and were secured with tape and a Polar strap over the handle of a Help'EmUp harness (Figure 1). This setup allowed for simultaneous recordings of HR with both systems while also allowing auscultation. The HOLTER monitor provided ECG tracings of HR over time. The HR MONITOR provided a constantly updating number indicating the current HR in beats per minute on its visual display and did not display or record an ECG-like tracing. The experiment occurred in a quiet room with a temperature between 18 and 22°C. Dogs were fasted for at least 2 h before the experiment.

### Heart Rate Recordings

**Figure 2:**
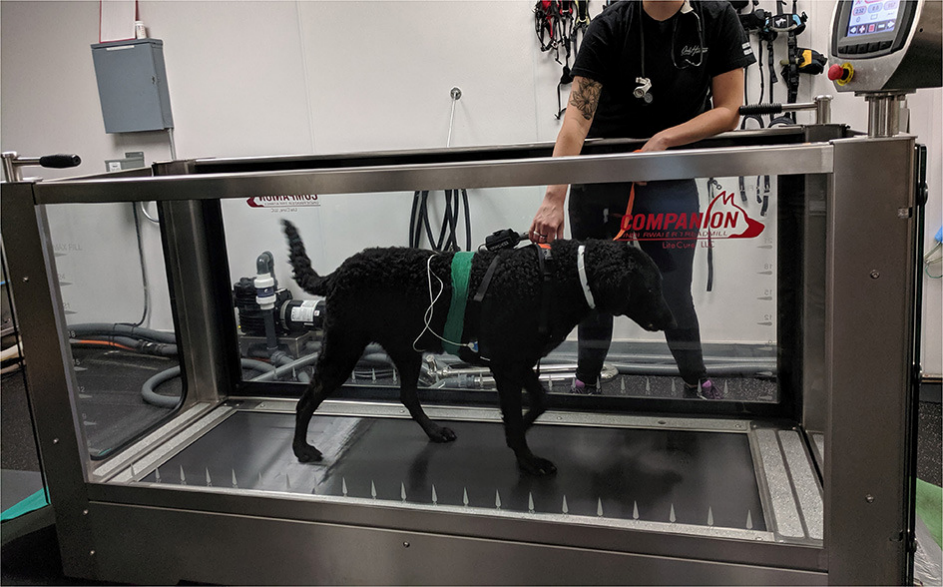
Dog equipped with both monitors while on the treadmill.

The treadmill (Companion Underwater Treadmill, Ft. Wayne, IN, USA) used for this study had no water present and 0 degrees of incline (Figure 2). HR was recorded in 1-min time intervals before TE after being in the treadmill room for 5 min (REST), immediately before TE while inside the non-moving treadmill (PRE), while walking on the treadmill (WALK) for 4 min, and 15 s (POST-15) and 60 s following TE (POST-60). The same individual who auscultated the dogs did so from the same position outside of the treadmill for the PRE, WALK, and POST-60 timepoints. Each of the TE sessions was 6 min in duration with a speed of 0.67 m/s (for a total of 239.57 m). This speed was selected since it is the speed used in the rehabilitation practice for any patient of this size who has TE as part of their therapeutic regimen. Dogs completed this protocol three times in 1 day.

All three HR monitoring methods were used for REST, PRE, and POST-60. Only the HOLTER and HR MONITOR collections were accomplishable while walking (due to the noise from the fur movement under the stethoscope bell obscuring the heart sounds) and were recorded at POST-15. Real-time video recordings were made of the screens of both monitors. HR was auscultated over the course of a minute to count the heartbeats, as the HR obtained over 15 s has been reported to have a 2–5 bpm difference from HR counted over 60 s (48, 49). HOLTER transmitted the HR data *via* Bluetooth to a nearby laptop. The HR MONITOR transmitted the data *via* Bluetooth to a nearby iPhone 7S. HR from the HOLTER was determined by manually counting the QRS waves recorded in CardioExplorer (Dextronix, Sacramento, CA, USA). For the HR MONITOR, the numbers present on the video recording of the iPhone screen at 0, 15, 30, 45, and 60 s during the minute of interest were averaged to determine the HR.

### Statistical Analyses

Statistical analyses included the Shapiro-Wilk test for normality assessment, Spearman or Pearson correlation coefficients [with a correlation of > 0.7 being desirable (22)], Bland-Altman plots with 95% limits of agreement (the HOLTER monitor, which is based on ECG recordings, was considered as the gold standard) to investigate bias (means/difference), and repeated measures ANOVAs with Sidak's *post-hoc* test (significant at *p* < 0.05). An *a priori* sample size of eight dogs was determined with an alpha of 0.05, 80% power, a mean difference of 20 beats, and a SD of 10 (http://openepi.com/Menu/OE_Menu.htm).

## Results

### The Effect of TE and HR Monitoring Feasibility

The mean (±SD) HRs for monitoring options at all timepoints are found in Table 1. Auscultation during exercise (WALK) was not possible due to the sound of hair movement muffling the heart sounds. TE significantly increased HR (mean increase of 20 bpm ± 4.3, *p* < 0.05) during WALK as compared to PRE, REST, POST-15, and POST-60 in both the HOLTER and the HR MONITOR (Table 1). Elevations in HR during WALK were not reflected in the POST-15 or POST-60 timepoints. Peak HR between the three WALK sessions was not significantly different (*P* = 0.29).

**Table 1:** Mean (±SD) HR of all monitoring options at different timepoints (*N* = 8 dogs).

**Monitoring option**	**REST**	**PRE**	**WALK**	**POST-15**	**POST-60**
Auscultation	102 (9)a	98 (8)a	N/A	N/A	98 (8)a
HOLTER	103 (10)a	96 (7)a	117 (1.5)b	103 (9)a	99 (8)a
HR MONITOR	103 (6)a	102 (15)a	121 (5)b	107 (14)a	108 (12)a

*HR during WALK was not possible to obtain with auscultation. Different letters indicate differences within a row between timepoints. N/A indicates not able to be obtained*.

The HOLTER could not capture instantaneous HR, which was known before the start of the study, and this did not affect our ability to upload the data and assess HR by counting the QRS waves in the proprietary software. The HR MONITOR would not always upload the HR during the sessions after their completion to the cloud, which was known before the start of the study and which was not an issue due to the screen video recordings.

### Comparison of HR Agreement for All HR Monitoring Options

Significant strong correlations existed between HOLTER and HR MONITOR at REST and WALK (both *rho* = 0.9, *p* < 0.01) and between the HOLTER and auscultation at REST, PRE, and POST-60 (all *rho* = 0.9, *p* < 0.01). There was a significant moderate correlation between HOLTER and HR MONITOR at PRE (*rho* = 0.75, *p* < 0.001) and POST-15 (*rho* = 0.72*, p* < 0.001). Auscultation and HR MONITOR moderately correlated at PRE (*rho* = 0.5, *P* = 0.01) and POST-60 (*rho* = 0.55, *p* < 0.001) but had a significant strong correlation at REST (*rho* = 0.9, *P* = 0.002).

**Figure 3:**
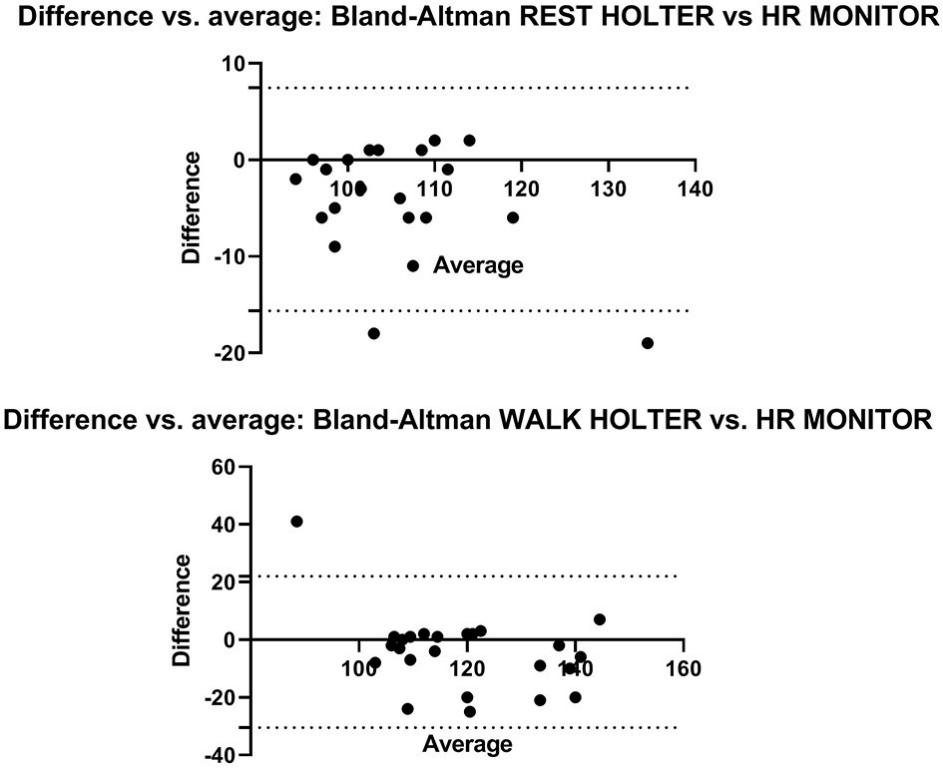
The Bland-Altman plots show the agreement between the HOLTER and HR MONITOR monitors during HR measurement at REST (top) and WALK (bottom). The X axis is the average of the HOLTER and HR MONITOR, and the Y axis shows the mean difference between the methods. The dotted lines represent the upper and lower 95% limits of agreement.

Bland-Altman analyses of HOLTER vs. HR MONITOR showed all but two data points at REST, and one data point at WALK was within the 95% limits of agreement (Figure 3), although there was a bias for the HR MONITOR to give higher HR readings than HOLTER during both REST (bias = −4.1) and WALK (bias = −4.2).

### Examination for Correlations Between Peak HR and Age and Peak HR and Weight

Peak HR did not statistically correlate with the age (*r*^2^ = 0.001, *P* = 0.94) or body weight of the dog (*r*^2^ = 0.14, *P* = 0.36).

## Discussion

The hypothesis that the HR could be readily measured before, during, and after a TE session using a HR monitor was accepted, noting that different monitors may be better for different applications. The hypotheses that HR is elevated during walk exercise as compared to rest in healthy dogs and that post-exercise HR measurements do not accurately demonstrate the HR achieved during exercise were accepted. Relying on post-exercise auscultation alone could underestimate workload, fear, and pain, leading to increased patient risk of injury.

Boddy et al. (20) and Swimmer and Rozanski (21) found no difference between pre-exercise HR values and post-exercise HR values in healthy dogs. Similarly, in the present study, no monitoring technique found a difference between the pre-exercise and post-exercise measurements. In Boddy et al. (20) any HR elevations during the exercise may have been missed as HR was assessed only once the dog stopped and not while the dog was still exercising. Swimmer and Rozanski (21) found that the dogs affected by pulmonary disease had an elevated post-exercise HR value despite walking less distance than normal dogs, but the true extent of HR elevation cannot be known as HR during exercise was not assessed. In a study that measured HR during a trot, Essner et al. (26) found an increase in HR during activity from baseline (124 ± 15.6 bpm from a baseline of 86 bpm). The current study also saw an increase in HR with walk exercise, though as expected the change in HR was less robust [20 bpm difference vs. ~38 bpm in the study of Essner et al. (26)] due to the slower gait. In the current study, the HR monitors did allow for HR measurement during TE (WALK), which was significantly higher than the rest, pre- and both post-exercise measurements. This would support a greater need to identify intra-exercise HR values and for the establishment of normal parameters in healthy animals.

The unexpectedly rapid return to pre-exercise HR levels could be due to the high vagal tone of healthy animals undergoing light exercise. A fast HR recovery at 10 s after cessation of exercise has been linked to lower mortality and is attributed to the reactivation of the parasympathetic nervous system while the sympathetic nervous system withdraws (45). In humans and dogs with cardiac disease, high-intensity interval training sped up HR recovery, and a faster HR recovery led to less risk for ventricular fibrillation (41, 42). Dogs with induced cardiac disease that underwent prolonged submaximal exercise regained higher vagal tone and had lower HRs post-exercise than sedentary dogs (43). It is possible that the healthy dogs in this study had higher levels of vagal tone due to their regular 4 days a week of brisk walk activity such that their return to baseline HR post-exercise was rapid. While athleticism can ensure a fast HR recovery, the intensity of exercise can override vagal tone. For instance, in racing greyhounds undergoing high-intensity exercise (a100 m sprint), the elevation of HR was seen in the immediate post-exercise auscultation (30). Most rehabilitation patients are not as fit as either the normal dogs in the current study or the racing greyhounds in the previously mentioned study. HR elevations during exercise and afterward would be predicted in all dogs, but the amount likely depends on the dog's health status and the type of exercise being performed.

Peak HR did not correlate with the age or the body weight of the dogs in this study. Previous work by Hezzell et al. (50) found that body weight, but not age, had a negative association with HR. A 50 kg decrease in weight was only associated with a 10.5 bpm increase in HR so it was not unexpected that in the current study, where the dogs were close in body weight, no association was seen. While age was significant in their initial univariate analysis, having a positive association with HR, it was not significant in their final model, nor was it significant in this study (50).

In this study, the investigators did identify some peculiarities in the management of the HR monitoring systems. During the study, the HR MONITOR software would not consistently upload *via* the cloud for storage and would not permanently store it. This was deemed a network and software issue that could be overcome. The HOLTER did not display instantaneous single-number HR values on the screen in real time. It was believed that this was a software setting that could be updated. Both of these issues were known prior to this study, and our methodology allowed for appropriate data collection and analysis. The data that were collected for both systems did correlate with the auscultated values collected. The harness and tape assembly used to secure the system of the dog was applied quickly with minimal effort, allowing the dogs to move freely. Thus, either of these monitors could be used to evaluate the HR during commonly performed therapeutic exercises, such as cavalettis and balance work or during underwater TE (the latter has been performed in our clinic with water at shoulder height with the HOLTER). Although it is possible submersion in the treadmill water could alter measured HR, the HOLTER monitor has already been used to investigate HR in diving seals (46, 47), suggesting that submersion in treadmill water would not be an issue.

Heart rate monitoring agreement was good to excellent between the methods and similar to previous reports (20, 21), indicating that either the HOLTER or HR MONITOR could be used in practice to provide insight into HR changes with walk exercise. The slight overestimation of HR in the HR MONITOR suggests that the HOLTER would still be preferred if available. Auscultation has been replaced by Holter monitoring of HR if possible in human research studies as the former has been found to be inaccurate (51). The limitations of this study included that only healthy, non-obese, large breed dogs were used and that the monitors were only assessed for HRs of up to 150 bpm at one speed. Normal HR variability and recovery with exercise should be investigated in medium and small breed dogs as has been done with swimming exercise (30).

Real-time HR monitoring appears to be feasible in rehabilitation practice, and the results of this study can offer some guidelines for use. While exercising at 0.67 m/s, a speed appropriate for walk rehabilitative TE, it was necessary to evaluate the HR during motion, as HR measurement post-activity did not reflect the elevated HR in healthy dogs while walking on a treadmill. Future studies at other paces, during other exercises, and in patients undergoing rehabilitation would be encouraged to record HR during the actual activity as well. The HOLTER, should allow updates for the projection of real-time instantaneous HR numbers on the monitor, which would be good for practice or research, but in its current form, it is more appropriate for research. The HR MONITOR would be appropriate for practice as it offers real-time instantaneous HR assessment, but is less than ideal for research as its software limitations require additional steps to analyze. Developing normal ranges for HR parameters during rehabilitation exercises can guide post-operative TE regimens to minimize patient risk of injury and to maximize the efficiency of recovery and training effectiveness. Exceeding a 20% increase in HR during walk TE in a large-sized dog could indicate that they are exceeding their conditioning (which could lead to fatigue and injury) or that they are under increased pain or physiologic stress which could be mitigated by adjusting the intensity or duration of the TE. Further study of HR during TE in populations of animals with various cardiovascular, musculoskeletal, and neurologic conditions is warranted.

## Data Availability Statement

The raw data supporting the conclusions of this article will be made available by the authors, without undue reservation.

## Ethics Statement

The animal study was reviewed and approved by Michigan State University's Institutional Animal Care and Use Committee. Written informed consent was obtained from the owners for the participation of their animals in this study.

## Author Contributions

SS contributed to research idea, study design, data collection and analysis, and manuscript preparation. SR contributed to study design, data collection and analysis, and manuscript preparation. RG and JM contributed to study design, data analysis, and manuscript preparation. All authors contributed to the article and approved the submitted version.

## Conflict of Interest

RG was employed by company Sportsvet Veterinary Consulting Services. The remaining authors declare that the research was conducted in the absence of any commercial or financial relationships that could be construed as a potential conflict of interest.
